# Ion currents through the voltage sensor domain of distinct families of proteins

**DOI:** 10.1007/s10867-023-09645-z

**Published:** 2023-10-18

**Authors:** César Arcos-Hernández, Takuya Nishigaki

**Affiliations:** Departamento de Genética del Desarrollo y Fisiología Molecular, Instituto de Biotecnología, Universidad Nacional Autónoma de México, Cuernavaca, Morelos, 62210 Mexico

**Keywords:** Voltage sensing domain, Omega current, Voltage dependent protein, Channelopathies, Gating pore current

## Abstract

The membrane potential of a cell (V_m_) regulates several physiological processes. The voltage sensor domain (VSD) is a region that confers voltage sensitivity to different types of transmembrane proteins such as the following: voltage-gated ion channels, the voltage-sensing phosphatase (Ci-VSP), and the sperm-specific Na^+^/H^+^ exchanger (sNHE). VSDs contain four transmembrane segments (S1–S4) and several positively charged amino acids in S4, which are essential for the voltage sensitivity of the protein. Generally, in response to changes of the V_m_, the positive residues of S4 displace along the plasma membrane without generating ionic currents through this domain. However, some native (e.g., Hv1 channel) and mutants of VSDs produce ionic currents. These gating pore currents are usually observed in VSDs that lack one or more of the conserved positively charged amino acids in S4. The gating pore currents can also be induced by the isolation of a VSD from the rest of the protein domains. In this review, we summarize gating pore currents from all families of proteins with VSDs with classification into three cases: (1) pathological, (2) physiological, and (3) artificial currents. We reinforce the model in which the position of S4 that lacks the positively charged amino acid determines the voltage dependency of the gating pore current of all VSDs independent of protein families.

## Introduction

### Proteins with voltage sensor domain

**Fig. 1:**
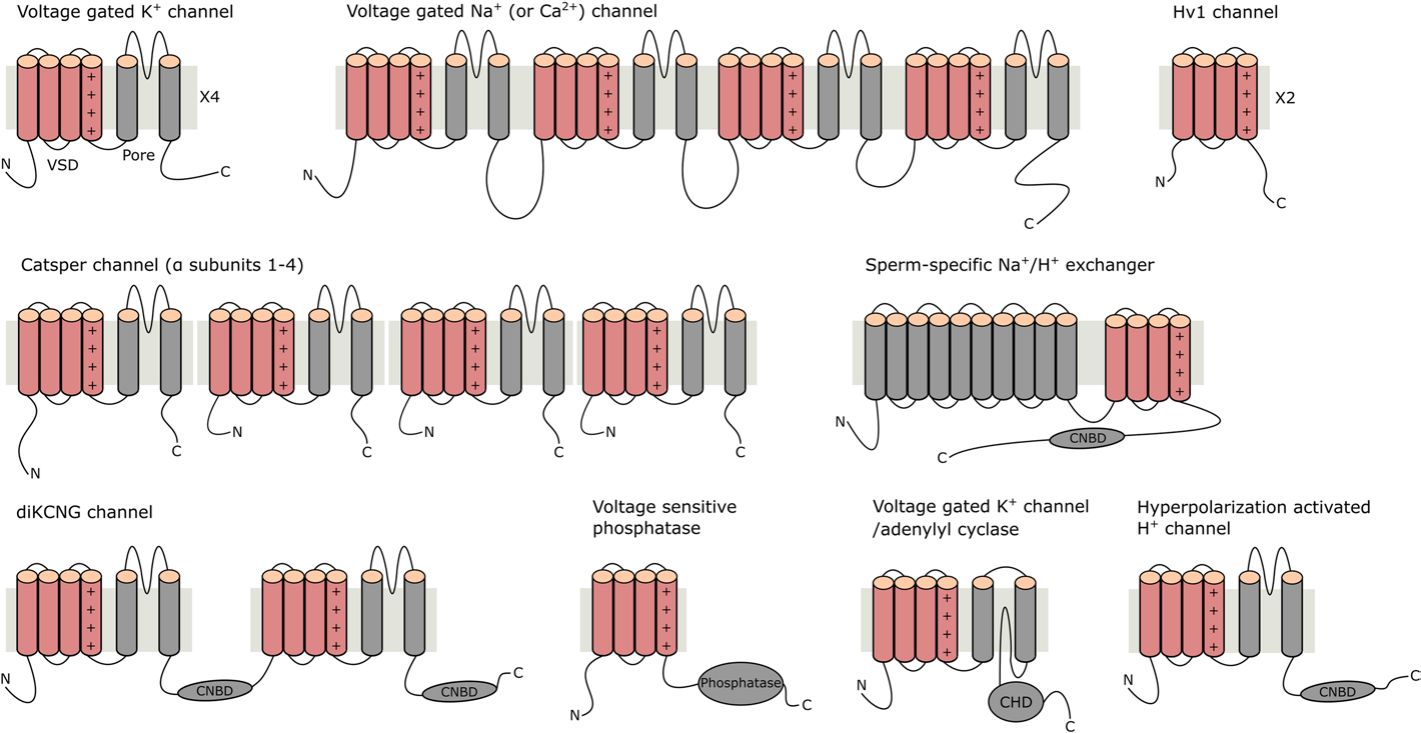
Diversity of proteins with a voltage-sensing domain (VSD). Topology of all the proteins reported until today that contain a VSD. The VSD is colored in pink, and all the other protein domains are colored in gray. CNBD cyclic nucleotide-binding domain, CHD cyclase homology domain

The electric potential inside of a live cell is normally different from that outside the cell. The difference of electric potentials across the plasma membrane of the cell is called membrane potential (V_m_). The V_m_ can be modified through the translocation of charges and the movement of dipoles. The activity of many transmembrane proteins is affected by changes in the V_m_ because they contain charged or polar side chains that are within the transmembrane electrical field and can be moved or reoriented as the V_m_ changes [1]. Among them, there are some groups of proteins that contain at least a voltage-sensing domain (VSD), which confers them with high voltage sensitivity. A typical VSD contains four transmembrane segments (S1–S4), and the fourth segment (S4) has several positively charged amino acids (lysines, arginines, and histidines) separated by two hydrophobic residues (Fig. 1). Because all 4 transmembrane segments of the VSD are α-helixes [2, 3], the positively charged amino acids of S4 align nearly in the same side forming a helical wire. The other segments (S1–S3) possess conserved polar and negatively charged residues that could establish electrostatic interactions with the positively charged residues of S4 or help create water-filled vestibules [4–6]. Currently, it is believed that S4 helically moves within the phospholipid bilayer upon V_m_ changes [1, 7]. Actually, large and rapid movement of S4 across the membrane can be measured as gating currents of VSDs [8, 9].

There are several types of proteins with a VSD (or VSDs). The best known is the voltage-gated ion channel [10]. A voltage-gated K^+^ channel composed of 6 transmembrane segments (S1–S6) represents a general feature of all these channels (Fig. 1): the first 4 segments (S1–S4) form a VSD, and the last two segments (S5 and S6) with the extracellular loop form a pore domain (PD). For a functional K^+^ channel, PD needs to form a homo (or hetero) tetramer (Fig. 1). In contrast, voltage-gated Na^+^ or Ca^2+^ channels are composed of 24 transmembrane segments, 4 repeats of the 6 transmembrane units, in a single polypeptide (Fig. 1). Therefore, they intrinsically form a hetero tetrameric channel pore [11]. On the other hand, the sperm-specific Ca^2+^ channel named CatSper requires four separate and distinct polypeptides to form the Ca^2+^-selective channel pore [12, 13] (Fig. 1). In addition, there is another group of channels composed of two repeats of 6 transmembrane units named two-pore channels (TPC) [14] (Fig. 1). Recently, we reported that brown algae have channels structurally similar to TPC channel with typical K^+^-selective pore motives and possible cyclic nucleotide-binding domains (named diCNGK channel) [15].

Besides the voltage-gated ion channel, a functional VSD can be found in some other proteins. In 2003, Wang et al. reported a novel sperm-specific Na^+^/H^+^ exchanger (sNHE) as an essential protein to regulate mouse sperm motility [16]. As a distinct feature from other Na^+^/H^+^ exchangers, sNHE possess a VSD in the C-terminal of the catalytic domain (Fig. 1). The functional expression of the mammalian sNHE has not been achieved yet, but recently, the voltage dependence of the Na^+^/H^+^ exchange activity was unequivocally demonstrated using sea urchin sNHE expressed in CHO cells [17]. In 2004, a novel adenylyl cyclase containing a VSD and a K^+^ channel pore (Fig. 1) was cloned from protozoa such as *Plasmodium*, *Paramecium*, and *Tetrahymena* [18]*.* Although definite functional characterization of the recombinant protein has not been done yet, this protein should explain the production of cAMP upon V_m_ hyperpolarization in these microorganisms [19]. In 2005, a voltage-sensor-containing phosphatase of the ascidian *Ciona intestinalis* (Ci-VSP) (Fig. 1) was reported as a novel lipid phosphatase that is regulated by V_m_ [20]. Its voltage dependence was elegantly demonstrated through the activity of a Kir K^+^ channel, whose activity is dependent on phosphatidylinositol, but not V_m_. The VSD of Ci-VSP has been widely studied and extensively used to develop voltage-sensitive fluorescent indicators [21].

As the last example of a VSD-containing protein, a voltage-sensor domain–only protein (VSOP) [22] also known as voltage-gated proton channel (Hv1) [23] was identified in 2006. As its name stands for, VSOP/Hv1 is a VSD without any other functional domains, but this protein generates voltage-gated H^+^ current through the VSD by itself. Interestingly, a pH gradient across the membrane (∆pH) as well as V_m_ regulates the H^+^ channel activity by an unknown mechanism, which had been characterized very well prior to its molecular identification [24].

### Channelopathies

Channelopathies are disorders of ion channels (loss or gain of function) caused by mutations that can be inheritable. Channelopaties in Na^+^ channels (Na_v_s) are well known [25–27], but channelopathies in Ca^2+^ and K^+^ channels were also reported. A typical example of channelopathies is found in Na_v_1.1 channel with a decrease of the Na^+^ current and cellular excitability due to channel folding problems (loss of function). On the other hand, a mutation of the inactivation process of Na_v_1.1 channel can result in an opposite effect, namely establishing a constant current that leads to hyperexcitability (gain of function) [28, 29]. Similar phenomena were reported in Ca^2+^ channels like Ca_v_1.2 [30–33]. In the case of K_v_4.2 channel, a mutation in S6 (V404M) slows the activation of the channel and has been associated to autism and epilepsy. In another report, a mutation causing a truncated form of the same channel is associated with temporal lobe epilepsy, probably by affecting the trafficking to the membrane (loss of function) [34, 35].

As described above, mutations can affect the channel function in different ways. However, there is another type of channelopathies caused by mutations in positively charged amino acids located in S4 of the VSD that generate a leak current through the VSD, so-called, omega current (or gating pore current). In general, the omega current is observed when the channel is at rest. However, the omega current can be detected also during its activation. This type of current has been associated to several pathologies like epilepsy, arrhythmias, and periodic paralysis among others [36–47].

Historically, the omega current through VSDs can be observed in some VSDs with an undesired mutation, namely in a pathological situation (channelopathies), and/or non-physiological (experimental) conditions. On the other hand, occasionally, mutations in some VSDs are beneficial and might have acquired some physiological roles in some organisms such as VSOP/Hv1 channel.

**Fig. 2:**
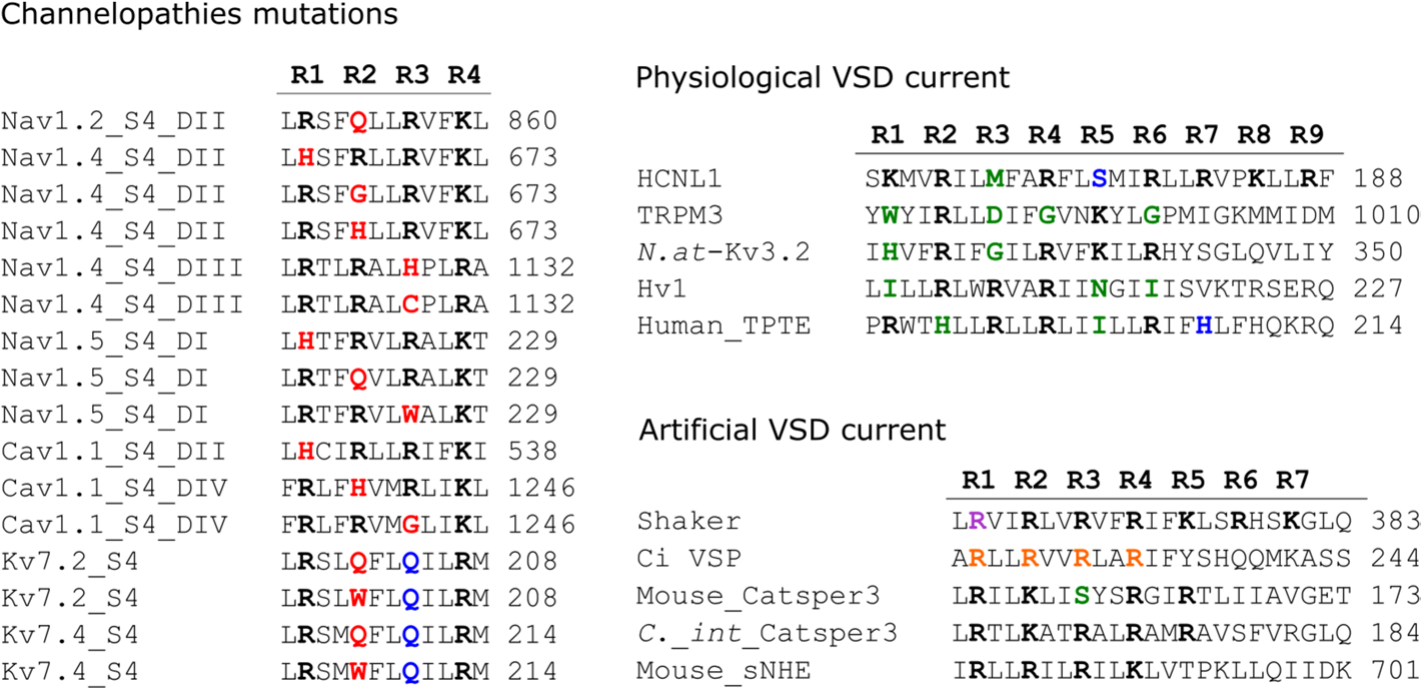
Positively charged amino acids and mutations in S4 of the VSD in proteins with an omega pore. Amino acid alignments of the S4 segment of the VSD that have been reported to exhibit gating pore currents. The proteins were divided into three groups depending on the origin of the omega pore. In the case of channelopathies mutations, the amino acids highlighted in red represent mutations of arginine residues. The amino acids highlighted in green represent positions in which other proteins contain positively charged residues. Conserved residues in certain proteins that are not positively charged amino acids are in blue. The positions R1 from Shaker channel in which mutations to C, S, A, and V results in omega currents are highlighted in purple. The positions R1 to R4 from Ci-VSP in which mutations to H results in omega currents are highlighted in orange. *N.at*-Kv3.2, Kv3.2 channel from *Notoplana atomata*; *C.*_*int*_Catsper3, Catsper3 subunit from *Ciona intestinalis*

In this work, we reviewed the omega currents (or gating pore currents) through the VSD of diverged proteins including our recent finding from the isolated VSD of mouse sperm-specific Na^+^/H^+^ exchanger (sNHE). We classified all omega currents into three categories: (1) pathological, (2) physiological, and (3) artificial currents. All of the mutations and specific characteristics of the omega currents mentioned in the next sections are summarized in Fig. 2 and Table 1.

**Table 1:** The mutations and specific characteristics of the omega currents

	**Channel or protein**	**Mutation(s), important amino acid or modification to overall structure**	**Physiological relevance or associated pathology**	**Type of current**	**Reference**
**Pathological VSD currents**	Nav1.2	R2Q (DII)	Idiopathic epileptic encephalopathies	Inward ion current activated by hyperpolarization	Mason et al. [47] eNeuro
	Nav1.4	-R1H and R2G/H (DII) -R3H/C (DIII)	Hipokalemic periodic paralysis	-Inward current at resting state -Outward current activated by depolarization (R3H/C)	Sokolov et al. [38] Nature Struyk and Cannon. (2007). JGP Gosselin-Badaroudine et al. [42] Proc. Natl. Acad. Sci Groome et al. [43] Brain
	Nav1.5	-R1H (DI) -R2Q and R3W (DI)	Cardiac arrhythmias and dilatation of cardiac chambers	-Proton-selective current activated by depolarization (R1H) -Outward K^+^/Inward Na^+^ current during AP. Inward Na^+^ current at resting state (R2Q, R3W)	Gosselin-Badaroudine et al. [42] PLoS ONE Moreau et al. [46] J. Gen. Physiol
	Cav1.1	R3G (DIV)	Normokalaemic periodic paralysis and recurrent cramping, oedema and neuronal compression with additional progressive myopathy	-Outward K^+^ current activated by depolarization. -Inward Na^+^ current activated by hyperpolarization	Fan et al. [45] Brain
		R1H (DII) and R2H (DIV)	Hipokalemic periodic paralysis	Inward cation current activated at resting state	Jurkat-Rott et al. [40] Proc. Natl. Acad. Sci Wu et al. [44] J Clin Invest
	Kv7.2 and Kv7.4	R4Q/R4W	-Benign familial neonatal seizures (BFNS) -Peripheral nerve hyperexcitability	Outward non-selective cation current activated by depolarization	Miceli et al. [41] Biophys. J.
**Physiological VSD currents**	Hv1	-	Many functions in several cell types: acid extrusion, volume regulation, acidification, etc	Outward proton current activated by depolarization and pH gradient	Sasaki et al. [22] Science
	TPTE (Human)	H207	Unknown	Outward proton current activated by depolarization	Sutton et al. [68] Mol. Biol. Evol
	Kv3.2	H325R, G331R	Unknown	Inward non-selective activated by hyperpolarization	Klassen et al. [67] BMC Neurosci.
	HCNL1	-	-Regulation of CNGK K^+^ channel activity -Zebrafish sperm activation of motility upon spawning into fresh water	Inward proton current activated by hyperpolarization	Wobig et al. [73] PNAS
**Artificial VSD currents**	Shaker VSD	Isolated VSD expression	_	Constitutively open channel at HP = −90 mV Inward non-selective cation currents at hyperpolarized potentials from a 0 mV HP	Zhao and Blunck. [90] eLIFE
	Ci-VSP	R1H, R2H, R3H, R4H	_	Outward proton current activated by depolarization	Villalba-Galea et al. [69] JGP
	Shaker	R1C, R1S, R1A, R1V	_	Inward proton current at resting state activated by hyperpolarization	Starace and Bezanilla. [85] Nature
	Catsper3 (*Ciona intestinalis* and *Mus musculus*)	Isolated VSD and whole subunit expression	_	Inward monovalent and divalent cation current activated by hyperpolarization	Arima et al. [95] BBA Biomembranes; Arima et al. [96] Channels
	Sperm-specific Na^+^/H^+^ exchanger (sNHE)	Isolated VSD expression	_	Outward ion current (selectivity not determined) activated by depolarization	Arcos-Hernández et al. [98] Protein. Expr. Purif.

## Pathological gating pore current

### Na_v_ channels

In 1999 and 2000, two groups identified mutations in the VSD of the second domain (DII) of Na_v_1.4 channel in families with hypokalemic periodic paralysis (HypoPP). HypoPP is an autosomal dominant disease characterized by attacks of flaccid paralysis and hypokalemia (lower than normal serum levels of K^+^). These mutations corresponded to specific arginines (R1 and R2) within the S4 of the VSD [48, 49]. However, at that time, the exact consequence of the mutations on the channel activity was not determined. In 2005, using a heterologous expression system, an omega current in rat Na_v_1.2 was recorded when R1 and R2 in S4 (DII) were mutated to glutamine [50]. These results suggest that HypoPP individuals with similar Na_v_1.4 mutations might be caused by an omega current as well. This hypothesis was confirmed in 2007 with a heterologous expression system, namely, Na_v_1.4 gene mutations (R1H and R2G/H DII) generated the omega current [38, 39]. In these reports, it was confirmed that the permeation of H^+^, K^+^, Na^+^, Cs^+^, Li^+^, and even larger ions like N-Methyl-D-glucamine (NMDG^+^) through the VSD depending on the position and identity of the mutated residues. In general, the omega current in these channels is proposed to increase the conductance at the resting membrane potential producing Na^+^ influx in skeletal muscle fibers. This leads to a more depolarized state that provokes inappropriate action potentials. This explanation coincides with the phenotype of a Na_v_1.2 mutant (R2Q DII) recently reported to be involved in human epilepsy cases [47]. Like the aforementioned observations, there is another report of a gating pore with certain different characteristics arising from mutations of R3 (H or C) of the third domain of Na_v_1.4. In these cases, an outward current activated at positive potentials alters the properties of action potentials. These mutations are also related to the inactivation of the channel and immobilization of S4. The current was recorded using various permeable cations like K^+^ and guanidinium. This produces an inward omega current that depolarizes the cell, which makes this mutant to have the same HypoPP phenotype seen with the other mutations [43].

In addition to Na_v_1.4 mutations related to HypoPP, some reports show that mutations in Na_v_1.5 VSD are also related to pathologies. In 2012, the mutation R1H (DI) was identified in a patient with arrhythmia and dilated cardiomyopathy (a genetic non-ischaemic heart muscle disease defined by left or biventricular dilatation and systolic dysfunction). This mutation generates a pH-dependent and depolarization-activated H^+^ current that promotes further depolarization and acidification of the cell [51]. Later, another depolarization-activated non-selective cationic current was reported in Na_v_1.5 mutants R2Q and R3W (DI) in which an impact on the canonical current through the pore domain was also found [46]. Even though these two mutants have different effects on the biophysical properties of the channel, both of them seem to be related to the same mentioned pathologies for R1H.

### Ca_v_1.1 channel

So far, there is only a single Ca^2+^ channel (Ca_v_1.1) in which certain mutations produce omega currents that are associated to pathologies. Two mutations of Ca_v_1.1 (R1H (DII) and R2H (DIV)) were found in several HypoPP patients, in which the severity of their symptoms was dependent on the extracellular K^+^ concentration probably due to a lower activity of the K^+^ inward rectifier channels [40]. The patients also had a more depolarized membrane potential of the myofibers. As happened with the Na_v_1.4 mutations, the more depolarized membrane potential was caused by a Na^+^ leak current at the resting membrane potential that results in reduced membrane excitability and muscle weakness [40]. Later, the phenomenon recorded in patients was studied using fibers from a mutant model mouse for Ca_v_1.1 (R1H (DII)) in which the same symptoms and a smaller La^3+^ sensitive cation current in comparison to the WT were described [44].

On the other hand, there is another mutation in Ca_v_1.1 channel (R3G (DIV)) that leads to two types of cationic omega currents, one with an outward direction that directly alters the form and size of action potentials causing hypoexcitability of the muscle and weakness. The other has an inward direction, depolarizing the cell and promoting Ca^2+^ release in the absence of action potentials, therefore causing aberrant muscle contraction. These and other symptoms are accompanied by normokalaemic periodic paralysis (NormoPP; paralysis associated to normal levels of K^+^ in serum) that are probably maintained by a K^+^ efflux through the omega pore and delayed rectifier channels. This mutation was identified in members of an American family presenting the aforementioned symptoms [45].

### K_v_ channels

Since 1998, mutations in K_v_7.2 channel have been associated to benign familial neonatal seizures (BFNS; an autosomal dominant syndrome characterized by seizures that start during the first week of life) [52, 53]. In accordance with this, in 2012, a group recorded the activity of the K_v_7.2 channel and the structurally and functionally related channel K_v_7.4 carrying the same mutations of the fourth segment of the VSD (R4Q and R4W). In both cases, the mutations generated a cationic omega current at depolarized potentials. Interestingly, there are two reports in which the same mutations are the cause of skeletal muscle myokymia (spontaneous or repetitive fat muscle contraction as a result of nerve hyperexcitability) after BFNS (in several members of a family; R4Q) [54] or in the absence of BFNS (in a different patient; R4W) [55]. So, it is very likely that these symptoms are produced by the initiation of an omega current during the VSD activation, which probably provides the cell with a persistent depolarization that promotes hyperexcitability and myokymia [41]. It is also interesting that the mutation R1Q in some patients with infantile spasms and hypsarrhythmia only changed the current activation to more hyperpolarized potentials but did not produce any omega current [56].

## Physiological gating pore current

So far, we have talked about the omega current as the cause of different pathologies; however, that is not always the case. The proteins mentioned in this section have naturally evolved to produce gating pore currents that participate in several physiological processes like the regulation of pH and membrane potential.

### H_v_1 channel

H_v_1 is a proton-selective channel that opens in response to the depolarization of membrane potential and cytoplasmic acidification (Sasaki, Takagi, and Okamura 2006; Ramsey et al. 2006). It participates in several biological processes: bioluminescence in dinoflagellates [57], ROS production [58], sperm physiology [59], among many others. The participation in these processes is exerted by the regulation of intracellular pH or membrane potential. A unique property of Hv1 is that its voltage sensitivity is tightly regulated by pH gradient across the membrane. The VSD is the only and unique domain of Hv1 and has a particular sequence comprising three arginines in S4 (R1–R3) and other important acidic residues (in segments S1–S3) that establishes a structure capable of transporting protons [60]. Specifically, there is an aspartic acid (Asp112) and an arginine (Arg211) that are proposed to be the selectivity filter because mutations of these two residues result in the loss of ion selectivity [61, 62]. Certain polar residues in S1–S3 participate in the stabilization of either the closed or the open state. Also, VSD relaxation can stabilize the open state [63]. The model also suggests that changes in pH alone can regulate the activation of the channel by promoting the protonation of a different set of acidic residues [60]. This hypothesis is supported by recent reports that show that the gating current’s voltage sensitivity is dependent on the difference of extracellular and intracellular pH (∆pH) [64] and that similar conformational changes of the VSD, measured using patch clamp fluorometry, are induced either by ∆pH or membrane potential [65]. Ongoing investigations of H_v_1 are being made by many research groups in order to describe its exact biophysical properties and its role in many physiological events.

### N.at-K_v_3.2 channel

Another interesting example of an omega current was reported in K_v_3.2 channel from the platyhelminth, *Notoplana automata* (*N.at*-K_v_3.2). This K_v_ channel produces an unusual inward rectifier current, but it can also be a delayed rectifier channel with some specific mutations in S4 of the VSD [66]. This happens because the channel contains two functional pores: a gating pore in the VSD and the canonical pore of voltage-gated K^+^ channel. The gating pore is characterized by the presence of a histidine and a glycine in the first and second positions that are usually occupied by positively charged amino acids in S4. This characteristic is important to produce a naturally occurring non-selective cationic inward omega current at hyperpolarized potentials that coexists with the K^+^-selective outward current through the pore domain activated at depolarized potentials. The typical current of K_v_3 channels can be obtained when the abovementioned histidine and glycine are mutated to arginines [67]. In spite that the biophysical characterization of *N.at*-K_v_3.2 has advanced, its physiological relevance remains unknown.

### Human TPTE

In 2005, a lipid phosphatase with a VSD from *Ciona intestinalis* was reported and called Ci-VSP (*Ciona intestinalis* voltage-sensitive phosphatase) [20]. Since then, its physiological properties have been progressively described, and the voltage sensor domain has been extensively used to produce several voltage-sensitive fluorescent indicators. The human orthologous gene of Ci-VSP is called TPTE (Transmembrane Phosphatase with Tensin Homology, also called Hs-VSP2). Due to the difficulty of expression of TPTE in a heterologous system, a chimera construction was produced between TPTE and *Danio rerio* VSP (Dr-VSP) to study the biophysical properties. When the chimera was expressed in HEK293 cells, a voltage-dependent outward H^+^ current was recorded. In this report, a histidine (H207 in the fourth segment of the VSD, indicated as R7 in Fig. 2) was proved to be an essential residue for the proton currents. The insertion of a histidine at the equivalent position in *D. rerio*’s orthologue (Dr-VSP) generated the same type of current, which suggests that the presence of histidine in this position is the key point to convert a VSD to H^+^ channel. Interestingly, TPTEs of most eutherian mammals conserve this histidine; however, rat and mouse have a glutamine instead of a histidine. This fact is curiously consistent with the lack of proton channel activity in mouse sperm. In human, H_v_1 channel is currently believed to form sperm voltage-gated H^+^ channel. However, it is possible that TPTE also contributes to voltage-gated H^+^ channel activity in human spermatozoa [68]. The orthologous protein Ci-VSP produces robust omega currents when any arginine in S4 is mutated to histidine [69]. Details were described in Sect. 4.2.

### HCNL1 channel

Hyperpolarization-activated and cyclic nucleotide-gated (HCN) channels are activated upon hyperpolarization of the membrane potential unlike most of the other voltage-gated ion channels [70]. They are structurally similar to K_v_ channels and are composed of 6 transmembrane segments (they form a homo-tetramer as a functional channel) with S4 having several arginine residues (Fig. 1). Also, its activity is modulated by cAMP through the CNBD found in the C-terminus of the channel. HCN has an ion-selective motif (GYGX) in the pore domain, similar to other K^+^ channels, but the fourth amino acid is not aspartic acid as other highly selective K^+^ channels (GYGD). Therefore, K^+^ selectivity of HCN channel is only about 4 times higher than Na^+^. As a consequence, HCN channels physiologically conduce a Na^+^ inward current and depolarize the cell, thereby regulating many biological processes like the electric activity in neurons [71] and sperm chemotaxis of marine invertebrates [72]. In 2020 a new hyperpolarization-activated channel from zebrafish sperm was described and called HCNL1 (HCN-like 1) [73]. Even though it has a high sequence identity to HCN channels, HCNL1 has very different characteristics. First, the canonical pore domain is not functional due to amino acid changes in the region corresponding to the selectivity filter of HCN, which has been proved to be essential for ion conduction [74]. The cyclic nucleotide-binding domain is also not functional in HCNL1 probably because an essential arginine for binding of cyclic nucleotides [75] is absent from the amino acid sequence. The functional pore is therefore located in the VSD, where a highly selective (similar to H_v_1) proton current is established. A methionine at the third position of the arginine sequence in S4 of HCN channels is an essential residue to the formation of the gating pore since its mutation to an arginine abolishes the proton current. These currents were recorded both in heterologous system (CHO cells) and in zebrafish sperm. The authors proposed that HCNL1 activity might be strongly related to that of the CNGK channel. The CNGK-induced hyperpolarization would activate HCNL1 provoking intracellular acidification that would block CNGK channel, establishing a negative feedback loop between these two proteins. The functional relationship between these two channels might be important for the activation of the motility of zebrafish sperm in fresh water. The inward H^+^ current through HCNL1 is observed at highly negative potential; therefore, this inward H^+^ current is functionally distinct from H^+^ outward currents through H_v_1 and human TPTE.

### TRPM3 channel

Transient Receptor Potential (TRP) channels are a large family of proteins that respond to numerous types of stimuli like pH, temperature, or ligand binding. In general, they exhibit weak voltage sensitivity (probably because their VSD lack most of the regularly spaced positively charged amino acids in S4) [76]. TRPM3 is part of the melastatin subfamily of TRP channels, and it can be activated by different compounds like the neurosteroid pregnenolone sulphate (PS), nifedipine, and the synthetic ligand CIM0216 along with changes in temperature and osmolality [77–80]. The channel has been linked to muscle contraction and Ca^2+^-induced insulin release along with the detection of noxious heat [77, 79, 81]. In 2014, an alternative pore was described for TRPM3. It was shown in this and other report that the combined stimulation with PS and Clt (a widely used antifungal drug) and the sole application of CIM0216 can open the alternative pathway, characterized by an inwardly rectifying current, different from the typical outwardly rectifying phenotype [82]. In this report, the existence of an alternative pore was suggested by different characteristics of the inwardly rectifying activity: (1) the voltage dependence was different from the canonical channel activity, (2) resistant to Ca^2+^ desensitization, (3) less sensitive to block by La^3+^, and (4) resistant to mutagenesis of the pore domain. Later, a more detailed report showed that TRPM3 produces the gating pore currents, namely, mutagenesis of several residues of S1, S3 and S4 of the VSD eliminated the inwardly rectifying currents [83]. A group of three tyrosine residues in S1 are indispensable for the alternative pathway; however, it is not clear if these residues are important for the gating of the omega pore or if they participate in the binding of the agonists. On the other hand, the residues in S3 (two negatively charged amino acids: E941 and D964) and S4 (W982, D988, and G991 corresponding to the arginines R1, R3, and R4 of the VSD of Shaker channel shown in Fig. 2) might be essential for the alternative pathway, because introducing positively charged residues in S4 probably establishes electrostatic interactions with the negatively charged amino acids in S3 that block the omega pore. Like other channelopathies, the activation of the alternative pathway of TRPM3 might produce Na^+^ influx at the resting membrane potential, resulting in an exacerbate TRPM3-dependent pain.

## Artificial gating pore current

### Omega current through the VSD of Shaker K^+^ channel

The Shaker potassium channel was the first K_**v**_ family identified in *Drosophila*. Since its discovery, it has been constantly used as a model to study general features of potassium channels [3, 84]. Derived from several works in heterologous systems, the effect of a number of mutations and modifications to the channel has been characterized including the induction of omega currents which will be addressed in this section.

#### Mutated VSD

In 2004 and 2005, an ion current through Shaker VSD (VSD_Sh_) was reported in response to mutations to R1 in S4 of the VSD. This residue was mutated to different amino acids: alanine, cysteine, histidine, serine, and valine, and in every case, a leak current was found. This current is activated at hyperpolarized potentials and can be separated from the canonical current through the pore domain [85, 86]. In a later report, it was found that a double gap in the sequence of arginines in S4 is sufficient to induce an omega current [87]. Tombola and collaborators in 2007 proposed that the cationic pathway might include the interphase between S4 and the canonical pore domain; however, a more recent report using molecular dynamics proposes a different pathway that follows the movement of the S4 segment and matches the position of the conserved arginines [88, 89]. The use of the Shaker channel to study the particular characteristics of ion conduction through the VSD could be important to understand certain pathologies related to the appearance of these types of mutations.

#### Isolated VSD

VSD_Sh_ can also develop an omega current when the domain is isolated from the pore without any amino acid mutations. Actually, several properties of the VSD_Sh_ change when the pore is removed: the voltage dependence becomes shallower, the deactivation gets slower, and the domain enters a relax state when exposed to prolonged hyperpolarization. Even though the permeation pathway is expected to be similar to the one of the mutated VSD_Sh_, the different properties of the domain in the absence of the pore result in a different type of omega current. Also, the gating pore seems to have different selectivity in comparison to the mutated VSD_Sh_, since this pore has a strong preference for protons but can also permeate other cations as large as NMDG^+^. Interestingly, the current is sensitive to ZnCl_2_ which could suggest some kind of similarity between VSD_Sh_ and H_v_1 gating pore [90].

### Proton current through Ci-VSP

As mentioned earlier, the voltage-sensitive phosphatase (Ci-VSP) is one of the most extensively studied proteins with a VSD. A structural study revealed the mechanism of voltage sensing of the VSD [91]. In this study, the crystallographic structures of the VSD showed the interaction between four arginines in S4 and the acidic residues in S1–S3 in the down and up state. In 2013, a histidine scanning also confirmed the involvement of the conserved arginines in S4 during the process of voltage sensing [69]. When the arginines were mutated to histidine, a robust proton current was recorded in all cases. This indicates that these arginines are sensing residues that transit through a hydrophobic region that focuses the electric field and represents the barrier for the movement of these charges from the intracellular to the extracellular space.

### Ca^2+^ current through CatSper3

CatSper is the main calcium channel in the sperm and is crucial for sperm motility regulation. The channel is a complex that consists of four pore-forming subunits (CatSper1-4) and many accessory subunits (CatSperβ, CatSperγ, CatSperδ, CatSperε, CatSperζ, and EFCAB9). Mice that are null for CatSper are infertile because spermatozoa cannot exhibit hyperactivated sperm motility, a vigorous flagellar beat required for sperm penetration through the oocyte [12, 92–94]. Like Shaker, the isolation of CatSper3 subunit VSD from *Ciona intestinalis* resulted in the establishment of an omega pore. In this case, the VSD can permeate monovalent and divalent cations when expressed in HEK293 cells. Interestingly, the full Catsper3 subunit including the pore domain was able to conduct the cationic current although the N-terminus of CatSper3 was substituted by that of Ci-VSP to promote the expression. The canonical pore did not conduct ions because it was mutated at the selectivity filter (D247A) [95]. A similar divalent cation current through the VSD of CatSper3 subunit was also recorded using the mouse orthologue [96]. However, the physiological relevance of the omega current through CatSper3 in the two mentioned species remains unknown. Further investigation will be required to address this issue.

### Chloride-dependent current through the isolated VSD of sperm-specific Na^+^/H^+^ exchanger (sNHE)

**Fig. 3:**
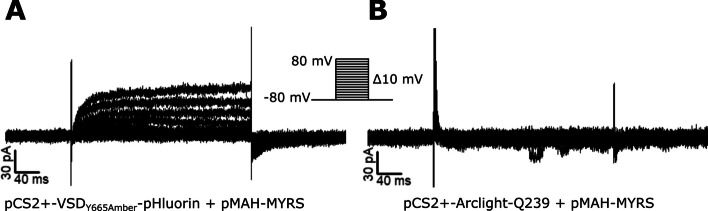
Ionic current through the isolated VSD of mouse sNHE. Current recordings of HEK293 cells co-transfected with pCS2 + -VSD_Y665Amber_-pHluorin and pMAH-MYRS (**A**) or with pCS2 + -Arclight-Q239 and pMAH-MYRS (**B**). The currents were obtained using a step protocol from −80 to +80 mV from a holding potential of −80 mV [98]. This figure was published in Protein Expression and Purification, Vol 201, César Arcos-Hernández et al, *How to study a highly toxic protein to bacteria: A case of voltage sensor domain of mouse sperm-specific sodium/proton exchanger*, Copyright Elsevier (2023)

The sperm-specific Na^+^/H^+^ exchanger (sNHE) is an essential protein for sperm physiology in mammals and invertebrates. The knock-out mouse for this protein is infertile, and mutations to sNHE in human patients have also been proved to affect fertility [16, 97]. A particular feature of this protein is the presence of two regulatory domains: a VSD and a cyclic nucleotide-binding domain [16]. Both of these domains were confirmed to modulate the catalytic activity in heterologous expression experiments using a sea urchin (*Strongylocentrotus purpuratus*) orthologue [17]. In our laboratory, we tried to determine if the isolated VSD of mouse sNHE was functional using electrophysiological techniques. The VSD resulted to be toxic to bacteria, and it was necessary to introduce an intron or a stop codon (that was suppressed later in mammalian cells) to obtain plasmids encoding the correct VSD. When we expressed the VSD in HEK293 cells, we observed an outward current that was not present in cells transfected with a VSD from a different protein (Fig. 3) [98]. So far, there are no reports of a similar outward current recorded in mouse sperm and the sea urchin sNHE expressed in CHO cells [17]. Thus, voltage-dependent outward currents observed from the VSD of mouse sNHE are probably an artifact of the isolation of the domain as it happens with the Shaker channel [90].

## Conclusions

**Fig. 4:**
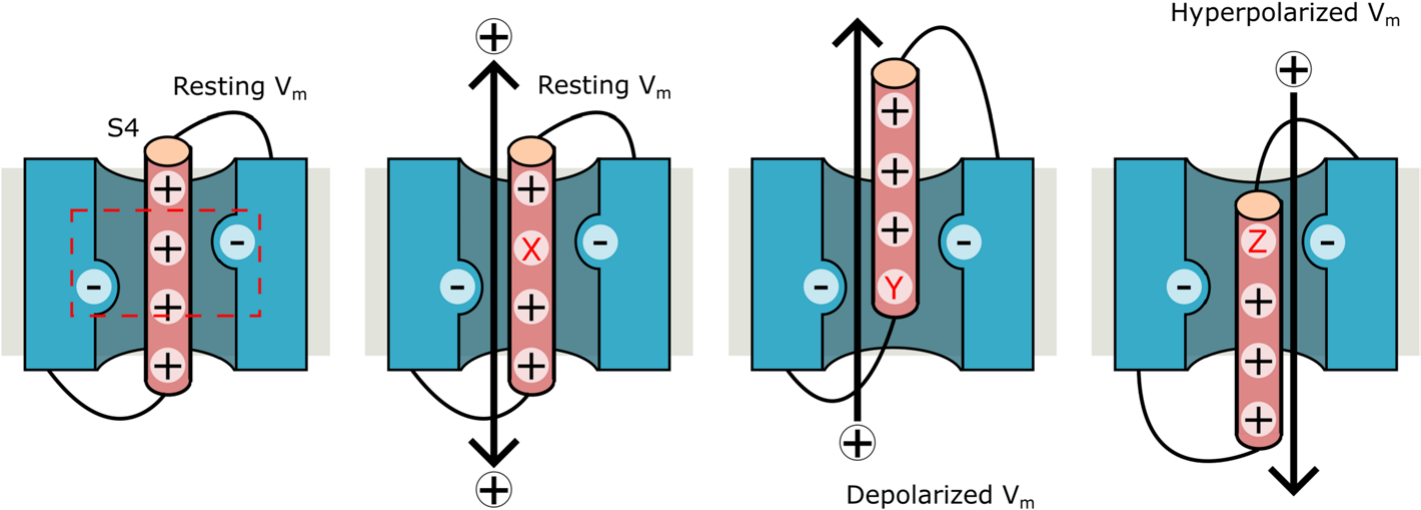
Gating pores and their voltage dependence. Gating pore currents are usually produced in VSDs through a discontinuous series of positively charged amino acids in S4. The position that lacks the positively charged amino acid (X, Y, and Z) determines the V_m_ value (resting, depolarized, or hyperpolarized) in which the gating pore currents will be generated. This happens because the tight interaction between the positively charged amino acids in the GCTC (discontinues red box) and the negatively charged or aromatic amino acids in S4 is loosened

Most VSDs of well-known voltage-gated channels contain S4 with 4–6 continuous positively charged residues (R or K) in every three amino acids that form a helical wire as described in the introduction. It has been proposed that S4 helically displaces through the gating charge transfer center (GCTC) according to a change of the membrane potential [85]. During the displacement of S4, the positively charged residues maintain tight interactions with negatively charged (or aromatic) residues of GCTC. Therefore, in our knowledge, there is no report of gating pore currents (omega current) from a VSD with such a typical S4 in physiological condition (correctly assembled proteins). In other words, all gating pore currents were observed from VSDs that have a S4 with discontinuous series of positively charged residues as we described in this review (Fig. 2). The position in S4 in which we find the discontinuity also seems to define the membrane potential range in which the gating pore currents will be provoked (Fig. 4). A similar model was previously proposed to explain the mechanism of gating pore currents focused on the VSDs of voltage-gated ion channels [99]. Thus, our review confirmed that their model is relevant in the VSDs independent of the function of proteins (not only voltage-gated ion channels but also voltage-activated lipid phosphatase and sperm-specific Na^+^/H^+^ exchanger). Therefore, the substitution of R (or K) by a certain amino acid seems a prerequisite to produce a gating pore current, but it is not a sufficient condition.

In this context, isolated VSDs of Shaker, mouse sNHE, and CatSper3 (without other CatSper subunits) are particular cases because they exhibit gating pore currents only in artificial condition without mutation in S4 or GCTC of other transmembrane segments. In these cases, no mutation was inserted in S4 of each VSD, but isolation from other interacting domains (or proteins) allows producing gating pore currents. We speculate that an isolation of VSD or subunit may provoke an abnormal movement of S4 of VSD, which may allow ion permeation through their VSDs.

There is significant diversity in amino acid substitution and the positions that generate gating pore currents. Also, biophysical properties of the currents such as ion selectivity, voltage dependence, and activation kinetics vary a lot depending on each case. Therefore, it is almost impossible to precisely predict or design a conversion from a normal VSD to a gating pore channel. However, future studies using protein structures might give us a better understanding of how the gating pores function, allowing researchers to develop, for example, molecules that effectively block the gating pores that cause diseases.

## Data Availability

Data sharing not applicable to this article as no datasets were generated or analyzed during the current study.
